# IDEAL CARDIOVASCULAR HEALTH STATUS AND HEALTH-RELATED QUALITY OF LIFE IN ADOLESCENTS: THE LABMED PHYSICAL ACTIVITY STUDY

**DOI:** 10.1590/1984-0462/2021/39/2019343

**Published:** 2020-08-28

**Authors:** César Agostinis-Sobrinho, André de Oliveira Werneck, Justina Kievišienė, Carla Moreira, Robinson Ramírez-Vélez, Rafaela Rosário, Sigute Norkiene, Luís Lopes, Jorge Mota, Rute Santos

**Affiliations:** a Faculty of Health and Sciences, Klaipeda University, Klaipeda, Lithuania.; b Universidade Estadual Paulista “Júlio de Mesquita Filho”, Presidente Prudente, SP, Brazil.; c Research Centre in Physical Activity, Health and Leisure, Faculty of Sport, University of Porto, Portugal.; d Department of Health Sciences, Public University of Navarra, Navarrabiomed- Pamplona, Navarra, Spain.; e School of Coimbra (ESEnfC), School of Nursing, University of Minho, Braga, Portugal.

**Keywords:** Health status, Physical activity, Diet, Cardiometabolic health, Youth, Estado de saúde, Atividade física, Dieta, Saúde metabólica, Jovens

## Abstract

**Objective:**

Adolescent’s ideal cardiovascular health index (ICVH) seems to be an important indicator of youth’s lifestyles and cardiometabolic health with potential positive consequences for their Health-Related Quality of Life (HRQoL). The purpose of this study was to examine the associations between the ICVH index and HRQoL in adolescents.

**Methods:**

This was a cross-sectional study based on secondary analyses from the LabMed Physical Activity Study (n=407 adolescents, 53% girls). ICVH, as defined by the American Heart Association, was determined as meeting ideal behaviors (physical activity, body mass index, smoking status, and diet intake) and health factors (blood pressure, total glucose, and cholesterol). HRQoL was measured with the Kidscreen-10 self-report questionnaire.

**Results:**

Analysis of covariance (ANCOVA) showed a significant association between the accumulation of ideal cardiovascular health metrics and HRQoL (F_(4,403)_=4.160; p=0.003). In addition, the higher the number of ideal health behaviors accumulated, the higher the mean values of HRQoL (p-value for trend=0.001), after adjustments for age, sex, socioeconomic status and pubertal stage.

**Conclusions:**

ICVH index was positively associated with HRQoL in adolescents. Ideal health behaviors metrics seem to have a stronger association with HRQoL than the ideal health factors metrics in adolescents.

## RESUMO

**Objetivo:**

O Índice de Saúde Cardiovascular Ideal (ISCI) do adolescente parece ser um importante indicador do estilo de vida e da saúde cardiometabólica dos jovens, com possíveis consequências positivas para a Qualidade de Vida Relacionada à Saúde (QVRS). O objetivo deste estudo foi examinar as associações do ISCI e da QVRS em adolescentes.

**Métodos:**

Este foi um estudo transversal baseado em análises secundárias do estudo *LabMed Physical Activity* (n=407 adolescentes, 53% meninas). O ISCI, conforme definido pela *American Heart Association*, foi determinado como atendendo a comportamentos ideais (atividade física, índice de massa corporal, tabagismo e padrão alimentar) e fatores de saúde (pressão arterial, glicose total e colesterol). A QVRS foi mensurada com o questionário de autorrelato Kidscreen-10.

**Resultados:**

A análise de covariância mostrou associação entre o acúmulo de métricas ideais de saúde cardiovascular e a QVRS (F_(4,403)_=4,160; p=0,003). Quanto maior o número de comportamentos ideais de saúde acumulados, maiores os valores médios da QVRS (p-valor de tendência=0,001), após ajustes para idade, sexo, status socioeconômico e estágio pubertal.

**Conclusões:**

O índice de saúde cardiovascular ideal associou-se positivamente à QVRS em adolescentes. As métricas ideais de comportamentos de saúde parecem ter uma associação mais forte com a QVRS do que as métricas ideais de fatores de saúde em adolescentes.

## INTRODUCTION

With the advancement of epidemiological transition, chronic diseases have become the greatest cause of death. Cardiometabolic disorders in adolescents are occurring at progressively younger ages worldwide and cardiovascular risk factors such as low HDL-cholesterol, elevated triglycerides, and high blood pressure are prevalent is adolescents.^1^ In 2010, the American Heart Association created a useful tool for cardiovascular risk surveillance called the Ideal Cardiovascular Health Index (ICVH index). This index comprises health behaviors (tobacco use, physical activity, body mass index, and diet) and health factors (blood pressure, plasma total cholesterol, and fasting glucose).^2^

Poor scores on the ICVH index have been associated with several negative health outcomes, such as inflammation,^3^ poor cardiac structure and function,^4^ and arterial stiffness^5^ during adolescence. Moreover, not meeting the ICVH index during childhood and adolescence has also been shown to be prospectively associated with cardiovascular outcomes in adulthood, such as metabolic syndrome, hypertension, and arterial thickness.^6^ However, the association between ICVH and psychological parameters are not well stablished.

Health related quality of life (HRQoL) is composed of indicators like social relationships, social support, depressive moods, and perception of cognitive performance.^7^ Assessing a youth’s HRQoL can help detect early impairments in well-being and functioning, as well as identify the subgroups of the population at higher risk for health problems.^8^ Having in mind the association between psychological/mental factors and metabolic risk,^9^ ^,^ ^10^ ^,^ ^11^ Pulkki-Råback et al.^12^ found that psychological factors during childhood/adolescence are associated with ICVH during adulthood. However, associations between ICVH and HRQoL have not been established among adolescents. In this sense, exploring the association between HRQoL and ICVH should support possible interventions on a broader range of variables aiming to improve ICVH, such as psychological factors, as well as better understand the association between psychological and biological risk factors among adolescents. We used data from the LabMed Physical Activity Study to examine the associations of HRQoL with ICVH index in adolescents aged 12 to 18 years.

## METHOD

Data for the present study was derived from the “Longitudinal Analysis of Biomarkers and Environmental Determinants of Physical Activity (LabMed Physical Activity Study)”, a school-based cohort study carried out in the North of Portugal. Selection of schools was based on pragmatic, budgetary, and logistical reasons. The full description of the study have been made elsewhere.^13^ In short, baseline data was collected in 2011 for 1,229 adolescents aged 12 to 18. The study participants’ recruitment was conducted at the selected schools. The students belonging to the 7th and 10th grades classes were invited to participate in the study. Of all the participants that agreed to participate in the LabMed study, 534 accepted to undergo blood collection, of those, 407 adolescents aged 12 to 18 years on baseline (218 girls) had complete data on the variables of interest for the present study. Power analysis was calculated post hoc (for α=0.05) and it was higher than 0.8 for analysis of covariance.

The adolescents and their parents or guardians filled the written informed consent, in agreement with the World Medical Association’s Helsinki Declaration for Human Studies. The Portuguese Data Protection Authority (#1112434/2011), the Portuguese Ministry of Science and Education (0246200001/2011) and Faculty of Sport, University of Porto, approved the study. Considering potential refusals to participate in the study due to blood analysis a “layered consent” was permitted. This allowed the participants to consent some parts of the study protocol and not others. For example, an adolescent could perform physical fitness assessments and refuse to undergo blood sampling. All adolescents whose parental and individual consents were received were enrolled in the study. Throughout the study, no exclusion criteria were applied to avoid discriminations. Nonetheless, we considered only apparently healthy adolescents, that is, without any medical diagnosis of physical or mental impairment.

The American Heart Association released the ideal cardiovascular health index in 2010^2^ with cut-off values for adolescents. Three health factors (blood pressure, total cholesterol, and fasting blood glucose) and four health behaviors (BMI, smoking behavior, physical activity, and diet) were considered for the ICVH metrics

Blood samples were obtained from each subject early in the morning by venipuncture from the antecubital vein, following a 10-hour overnight fasting. The samples were stored in sterile blood collection tubes at 4° to 8°C for no longer than four hours during the morning of collection and then sent to an analytical laboratory for testing according to standardized procedures. Fasting serum glucose concentrations were analyzed enzymatically, Hexokinase method (Siemens Advia 1600/1800 Erlangen, Germany). Total cholesterol CHOD-POD enzymatic method (Siemens Advia 1600/1800). All assays were performed in duplicate according to the manufacturers’ instructions and none of the study youths were on any drug treatments.

Ideal total cholesterol has been defined as “ideal” with values <4.40 mmol/L (<170 mg/dL), or “non-ideal” ≥4.40 mmol/L (≥170 mg/dL).^2^ Ideal fasting blood glucose concentrations were classified as ideal <5.6 mmol/L (<100 mg/dL), or non-ideal ≥5.6 mmol/L (≥100 mg/dL).^2^

Resting blood pressure was measured using a Dynamap vital signs monitor (model BP 8800, Critikon, Inc., Tampa, Florida). Trained nurses took measurements, and all adolescents were required to sit and rest for at least five minutes prior to the first blood pressure measurement. Participants were in a seated, relaxed position with their feet resting flat on the ground. Two measurements in the non-dominant arm were taken, after five and ten minutes of rest. The mean of these two measurements was considered. If both measurements differed by 10 mmHg or more, a third measure was taken. Blood pressure was defined as ideal (mean diastolic blood pressure or mean systolic blood pressure <90^th^ percentile) or non-ideal (mean diastolic blood pressure or mean systolic blood pressure ≥90^th^ percentile).^2^

Body height and weight were measured according to standard procedures with the participants lightly dressed and in bare feet with a portable stadiometer (Seca213, Hamburg, Germany) and a portable electronic weight scale (Tanita Inner Scan BC532, Tokyo, Japan), respectively. Body mass index (BMI) was calculated from the ratio body weight (kg)/body height (m^2^). Participants with a BMI <85^th^ percentile were categorized as meeting the ideal cardiovascular health criteria for BMI.^2^

Dietary intake and food consumption was assessed by the Kidmed questionnaire (Mediterranean Diet Quality Index for children and adolescents),^14^ an index ranging from 0 to 12 points. Participants were classified as having an ideal healthy diet (≥8 points), whereas children and adolescents with <7 points were classified as having a non-ideal healthy diet, according to previous studies.^15^

Physical activity was assessed with accelerometers GT1M (ActiGraph, Pensacola, Florida, USA). Participants were instructed to use the accelerometer attached on the right side of their hips, with the notch faced upwards, over five consecutive days (three weekdays, two weekend days) during waking hours, and remove it during water-based activities. The epoch length was set to 2 seconds to allow a more detailed estimate of physical activity intensity. Accelerometer data were analyzed by an automated data reduction program (ActivLive software v. 6.12, ActiGraph, Pensacola, Florida, USA). Periods with 60 minutes of consecutive zeros were detected and flagged as non-wear time. The cut-off points proposed by Evenson, Catellier et al. were used to determine physical activity intensities. Adolescents who performed, on average, more than 60 min of moderate-to-vigorous physical activity per day were classified as having an ideal physical activity level.^2^

Data on tobacco use was gathered with a self-reported questionnaire. Never-smokers were classified as having an ideal smoking behavior.^2^

The HRQoL was evaluated by KIDSCREEN-10, which consists of a 10-item scale. HRQoL was assessed using the Portuguese self-reported version of the KIDSCREEN-10 questionnaire,^16^ which has been transculturally developed in 13 European countries for the population of children and adolescents aged eight to 18 years. This instrument assesses ten dimensions and is used to validate evidence to support inferences about general measures of quality of life. KIDSCREEN-10 is a reduced version of the KIDSCREEN-52 questionnaire, which contains ten items assessed on a five-point Likert scale ranging from 1 (never; not at all) to 5 (always; extremely). KIDSCREEN-10 results in an overall value for quality of life. A low value in this tool suggests a feeling of dissatisfaction and inadequacy in many areas of children’s and adolescents’ lives, in particular, family, peer group and school. A high value, suggests a perception of adequacy and satisfaction with their contexts.^16^

Participants self-assessed their pubertal stage of secondary sex characteristics (breast and pubic hair development in girls and genital and pubic hair development in boys) ranging from stage I to V, according to the criteria of Tanner and Whitehouse.^13^

Adolescents’ socioeconomic status was assessed by the Family Affluence Scale,^13^ a four-item questionnaire that helps students report their family income objectively. The answers of the questionnaire were summed, and a continuum variable was computed to perform the statistical analyses.

Data are presented as means±standard deviation (SD) and percentages. Two-tailed Student’s t-test and chi-square test were used to test differences between boys and girls, for mean values and percentages, respectively. Differences of HRQoL scores between ideal and non-ideal cardiovascular health components were assessed with analysis of covariance (ANCOVA) with HRQoL score as a dependent variable, cardiovascular health component (ideal vs non-ideal) entered as a fixed factor, and age, sex, pubertal status, socioeconomic status as a covariate. ANCOVA was also used to study the differences of HRQoL score by ideal cardiovascular health metrics as well as ideal health behaviors and factors separately. HRQoL score was entered as dependent variable, the ideal cardiovascular health metrics as independent variable and age, sex, pubertal status, socioeconomic status as covariates. SPSS v.25 (IBM) statistical software was used to performed statistical analysis, and a p<0.05 denoted statistical significance.

## RESULTS

The characteristics of the participants are presented in Table 1. The prevalence of ideal cardiovascular health metrics was: non-smoking (91.2%), non-overweight (71.5%), physical activity (37%), diet (47.7%), total cholesterol (75.7%), blood pressure (90.9%), and plasma glucose (95.6%). Boys showed higher levels of moderate-to-vigorous physical activity than girls (p<0.05). Girls presented higher levels of total cholesterol (p<0.05).

**Table 1:** Participants’ characteristics at baseline.

	Total (407)	Girls (218)	Boys (189)
Age (years)	13.9±1.6	14.1±1.6	13.9±1.6
Systolic blood pressure (mmHg)	118.3±12.7	117.7±11.1	119.3±13.1
Diastolic blood pressure (mmHg)	63.3±7.8	64.1±7.5	62.7±7.8
Glucose (mg/dL)	88.8±7.1	87.6±7.4	89.8±6.3
Total cholesterol (mg/dL)	153.6±25.6	159.1±26.5	147.9±26*
Moderate-to-vigorous physical activity (min/day)	54.9±18.5	50.1±16.2	62.2±20.2*
Socioeconomic Status	6.5±1.6	6.4±1.7	6.5±1.5
HRQoL (Kidscreen-10)	39.5±5.3	39.1±5.5	40.1±5.2
Ideal health behaviors			
Nonsmokers (n)%	371 (91.2)	204 (93.6)	167 (88.5)
Non-overweight (n)%	291 (71.5)	155 (71)	136 (72.1)
Physically active (n)%	154 (37)	57 (26.1)	97 (51.1)*
Healthy diet (n)%	158 (47.7)	103 (47.2)	86 (45.5)
Ideal health factors			
Normal cholesterol (n)%	308 (75.7)	153 (70.2)	155 (82)
Normal blood pressure (n)%	370 (90.9)	198 (90.8)	172 (91)
Normal plasma glucose (n)%	389 (95.6)	208 (95.4)	181 (95.8)

*significantly different from girls (p<0.05).

**Figure 1:**
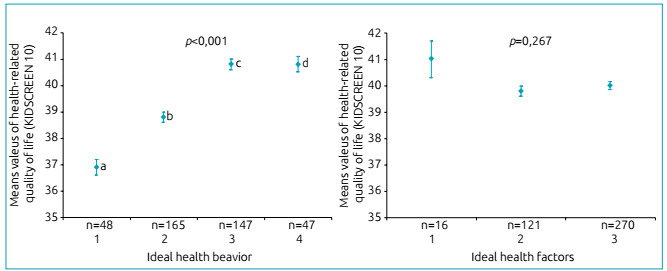
Mean values of health-related quality of life score by accumulated of ideal cardiovascular health metrics. Higher score was associated with a higher number of ideal cardiovascular health components (p-value for trend <0.001) after adjusting for age, sex, socioeconomic status and pubertal status.

ANCOVA showed significant association between ideal cardiovascular health metrics index and HRQoL score (F_(4,403)_=4.160; p=0.003), after adjustment for age, sex, socioeconomic status and pubertal status (Figure 1).

**Figure 2:**
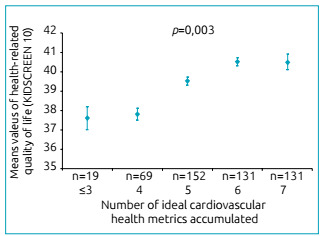
Health related quality of life score across the number of ideal health behaviors accumulated (smoking, body mass index, physical activity and diet) and the number of ideal health factors accumulated (total cholesterol, blood pressure and plasma cholesterol) score in adolescents, after adjusting for age, sex, socioeconomic status and pubertal status.

Figure 2 shows that a higher HRQoL score was associated with a higher number of ideal health behaviors (p for trend <0.001). Conversely, no significant association was found for HRQoL and ideal health factors (p=0.267).

## DISCUSSION

In this study, ideal cardiovascular health score was positively associated with HRQoL in adolescents. The accumulation of ideal cardiovascular health metrics was shown to be associated with a greater HRQoL score. In addition, we found a significant trend showing that the more ideal behavior metrics adolescents accumulated, the higher the HRQoL score.

Lifestyle behaviors are well stablished as protection factors for several mental disorders, even during adolescence and early adulthood. Ames and Leadbeater^17^ found that subjects with consistently higher numbers of depressive symptoms and/or an increased number of depressive symptoms in adolescence (12y to 18y) also presented lower levels of physical activity and greater rates of tobacco smoking, variables that compose ideal health behavior scores. In this sense, an ideal health behaviors score (smoking, BMI, physical activity, and diet) is associated with both mental and physical conditions, as well as symptoms of mental disorders and metabolic disorders, which are associated with a poorer HRQoL.^18^ ^,^ ^19^ ^,^ ^20^

We found a consistent association between ideal health behavior and HRQoL, but not for ideal health factors. HRQoL is generally understood as a multidimensional concept that consists of various components of well-being and functionality from the subjective perspective of the individual. In this way, HRQoL is associated with social integration factors, such as social support and perception of social relationships,^21^ ^,^ ^22^ which are also associated with mental disorders.^23^ Both social and mental factors compose the Kidscreen questionnaire. Beyond the social disintegration caused by risk behaviors, physical activity, tobacco smoking, and dietary patterns are also associated with changes in inflammatory and metabolic risk factors,^3^ ^,^ ^20^ which are associated with mental health outcomes as depressive symptoms and anxiety even during adolescence.^9^ ^,^ ^10^ ^,^ ^11^

In the present study we also found that even if ideal health factors were not individually associated with HRQoL, an aggregation of ideal health behaviors to obtain the ideal cardiovascular health showed a trend towards a better HRQoL score. Despite some studies addressing the association between ideal cardiovascular health and cardiovascular risk outcomes such as inflammation,^3^ cardiac structure and function,^4^ arterial stiffness,^5^ muscle fitness,^15^ cardiorespiratory fitness, to our knowledge, this was the first study to analyze the association between ICVH index and HRQoL in adolescents.

Another point considers the prevalence of ideal cardiovascular health metrics in adolescents. In the same line as our results, a recent study^24^ in European adolescents showed a low prevalence of ideal cardiovascular behaviors, especially for diet and physical activity. In another study, Shay et al.^25^ showed that approximately 47% of U.S. adolescents from the NHANES study had at least five ideal cardiovascular health components. They also showed that ideal physical activity and diet had the lowest prevalence. Collectively, these results (as well as ours) strongly motivate efforts to increase ideal health behaviors in this age group.

The association of HRQoL with ideal cardiovascular health is still not fully perceived in adolescents; however, some mechanisms have been proposed through greater social support,^22^ physical fitness,^15^ ^,^ ^26^ as well as through inflammatory mechanisms associated with both metabolic and mental risk factors.^3^ ^,^ ^9^ ^,^ ^10^ For instance, the pathways by which physical activity is associated with physical and psychological well-being can be explained by the fact that regular physical activity increases physical fitness and thus improves vascular and metabolic function,^27^ leading to favorable structural and functional neuronal adaptations^28^ and improved attention, emotions, inhibitory control, and academic performance.^29^ It all shows that HRQoL can serve as an important predictor for health status when compared to well-recognized risk factors. In a clinical setting (or schools), adolescents reporting poor physical HRQoL could be connected with health professionals to help improve their physical well-being, if intervention indicated. Furthermore, self-reported HRQoL is known to be representative of adolescent preferences and priorities, which is consistent within the school environment as a low-cost assessment to help identify youth at risk of poor health.^30^

The present study presents some limitations in its cross-sectional design, which does not allow causality inference to be drawn. Also, we objectively measured physical activity (with accelerometers), which gave reliable values of habitual physical activity but caused an elevated number of missing data due non-attended criteria and presented a possible reactivity effect. We used a valid questionnaire to determine the HRQoL.^7^ We also controlled our analyses for important covariates, such as age, sex, socioeconomic status, and pubertal status.

In conclusion, ideal cardiovascular health index was positively associated with HRQoL in adolescents. Ideal health behaviors metrics seem to have a stronger association with HRQoL than the ideal health factors metrics in this group. As ideal health behaviors were the mostly associated with HRQoL, intervention strategies focusing on increasing physical activity, healthier eating behaviors, and the prevention of obesity and tobacco use should be conducted.
